# Impact of Dendrimers on Solubility of Hydrophobic Drug Molecules

**DOI:** 10.3389/fphar.2017.00261

**Published:** 2017-05-16

**Authors:** Sonam Choudhary, Lokesh Gupta, Sarita Rani, Kaushalkumar Dave, Umesh Gupta

**Affiliations:** Department of Pharmacy, School of Chemical Sciences and Pharmacy, Central University of RajasthanKishangarh, India

**Keywords:** solubility enhancement, bioavailability, dendrimer, polyamidoamine, poly(propylene imine), polymer, solubilization, hyperbranched polymers

## Abstract

Adequate aqueous solubility has been one of the desired properties while selecting drug molecules and other bio-actives for product development. Often solubility of a drug determines its pharmaceutical and therapeutic performance. Majority of newly synthesized drug molecules fail or are rejected during the early phases of drug discovery and development due to their limited solubility. Sufficient permeability, aqueous solubility and physicochemical stability of the drug are important for achieving adequate bioavailability and therapeutic outcome. A number of different approaches including co-solvency, micellar solubilization, micronization, pH adjustment, chemical modification, and solid dispersion have been explored toward improving the solubility of various poorly aqueous-soluble drugs. Dendrimers, a new class of polymers, possess great potential for drug solubility improvement, by virtue of their unique properties. These hyper-branched, mono-dispersed molecules have the distinct ability to bind the drug molecules on periphery as well as to encapsulate these molecules within the dendritic structure. There are numerous reported studies which have successfully used dendrimers to enhance the solubilization of poorly soluble drugs. These promising outcomes have encouraged the researchers to design, synthesize, and evaluate various dendritic polymers for their use in drug delivery and product development. This review will discuss the aspects and role of dendrimers in the solubility enhancement of poorly soluble drugs. The review will also highlight the important and relevant properties of dendrimers which contribute toward drug solubilization. Finally, hydrophobic drugs which have been explored for dendrimer assisted solubilization, and the current marketing status of dendrimers will be discussed.

## Introduction

The role of aqueous-solubility of any new chemical entity (NCE) or a drug is crucial and decisive in the development of its formulation. While product development of aqueous-soluble drugs is relatively easy and less expensive, the formulation development of a drug with low aqueous-solubility consists of multiple challenging steps where solubility and solubilization of the drug substance has very high significance in the development process (Pace et al., 1999; Valentino and Kwame, 2007; Liu, 2008). According to published literature, majority of the drugs recognized through high-throughput screening techniques possess some solubility concerns (Wu and Benet, 2005; Guo et al., 2011).

A report published by Benet et al. (2011), which categorized 698 commercially available, orally administered immediate-release (IR) drugs using the Biopharmaceutics Drug Disposition Classification System (BDDCS), stated 33% of those drugs to be under BCS (Biopharmaceutical classification system) Class-II (i.e., high permeability and low solubility), and 6% of those drugs to be under BCS Class IV (i.e., having low permeability, low solubility) (Benet et al., 2011). The report estimated that only 5% of new molecular entities (NMEs) under development by industry had both high solubility and high permeability (BCS Class I), while another 5% were of BCS Class III. Approximately 70% of NMEs under investigation/development were BCS Class II compounds and 20% were BCS Class IV compounds (Benet et al., 2011). Though many of these entities are highly potent drug candidates, they are not taken further into the development due to their limited aqueous solubility. In the recent years several efforts have been made to solubilize hydrophobic drugs; some of those strategies are briefly discussed in the following section.

## Strategies for solubilization of hydrophobic drugs

### Solid dispersion

Solid dispersion is a process which involves at minimum two different solid components, generally a drug of hydrophobic nature and a hydrophilic matrix. Commonly used hydrophilic matrices are polyvinyl pyrrolidone (Povidone, PVP), polyethylene glycols (PEGs) and Plasdone S630. Solubility of poorly soluble molecules such as celecoxib and ritonavir was improved using solid dispersion through suitable hydrophilic carriers such as povidone (PVP) [for celecoxib] and gelucire [for ritonavir] (Abdul-Fattah and Bhargava, 2002; Gupta et al., 2004; Sinha et al., 2010). However, the use of this method has been limited due to fewer choices of suitable hydrophilic matrices forming stable solid dispersions.

### Nanosuspension

Nanosuspension is a biphasic system consists of nano-ranged particles stabilized using surfactant molecules, has been established as a potential strategy for the effective delivery of water insoluble APIs. The particle size range for nanosuspension is generally 200–600 nm (Muller and Peters, 2000).

### Particle size reduction

The solubility of a drug is fundamentally dependent on its particle size. As the size of drug particles decreases, the surface area increases leading to higher interaction of the drug particles with the medium; and hence improves the solubility of the compound (Blagden et al., 2007). The approach of particle size reduction has been applied in the past to drug molecules—such as griseofulvin and progesterone—for enhancing the solubility which led to improved digestive absorption, and consequently better bioavailability and clinical efficacy (Chaumeil, 1998; Vogt et al., 2008).

### Cryogenic approach

The method was established to improve drugs' solubility by developing highly porous nano-structured amorphous drug particles at very low temperature. After cryogenic processing, dry powder can be obtained through vacuum freeze drying (lyophilization) spray freeze drying, atmospheric freeze drying, etc. (Mumenthaler and Leuenberger, 1991; Leuenberger, 2002; Williams, 2003).

### Inclusion complex formation-based techniques

The inclusion complexes are designed through inserting a non-polar molecule or guest molecule inside the cavity of a host molecule (Uekama et al., 1998); Cyclodextrin is an example where the hydrophobic cavities provide a microenvironment for appropriate sized non-polar molecules and thus form drug cyclodextrin complex.

### Micellar solubilization techniques

In this approach, surfactants are used to enhance the drug solubility through reducing the surface tension, interfacial tension, solids wetting and the rate of disintegration (Edward and Li, 2008). Non-ionic surfactants such as polysorbates, polyoxyethylated castor oil etc. are commonly used for improving the solubilization of the drug (Rangel-Yagui et al., 2005; Hsu et al., 2008). Solubility of various anti-diabetic drugs such as gliclazide, glyburide and glipizide has been enhanced using the approach of micellar solubilization (Hsu et al., 2008).

### Supercritical fluid (SCF) process

One of the nanosizing and solubilization techniques which have been popular in recent years is supercritical fluid (SCF) process. SCF is a fluidic form of material whose temperature and pressure are beyond its critical point, which allows the material to possess the characteristics of both gas and liquid. When the material reaches near its critical temperature, it becomes very compressible making it susceptible to drastic changes in terms of its mass transport properties and density upon small changes in the pressure. When the material is in its supercritical region, its properties including surface tension, density, viscosity, solvency and diffusivity are intermediate to its gas and liquid states. Due to the unique properties, the solubilization capacity of the SCF significantly improves. Carbon dioxide is one of the popular SCF agents owing to its characteristics such as safety, cost-effectiveness, low toxicity profile, recyclability, and non-flammability (Girotra et al., 2013). When the drug material is solubilized in the SCF it recrystallizes in the form of uniform micron or nano-sized particles leading to its enhanced solubility (Sunkara and Kompella, 2002). The major limitations associated with SCF include the high cost and complexity of the operation (Girotra et al., 2013).

### Hydrotropy

In this method, a large amount of second solute (hydrotropic agent) is added to the solute intended for solubilization. The hydrotropic agents such as ionic, organic salts, which are composed of alkali metals or various organic acids, increases the water solubility of the first solute, due to greater quantity of additives. The mechanism behind the improvement in the solubility is the weak interaction between the hydrotrophic agent (such as sodium benzoate and sodium acetate) and the poorly soluble drug (Badwan et al., 1983; Rasool et al., 1991).

Limitations of the conventional drug solubilization strategies (Pace et al., 1999; Bachhav and Patravale, 2010) have led to exploration and invention of newer drug solublization techniques. Several colloidal and vesicular systems such as liposomes, microspheres, nanoparticles and microemulsions, have been studied for solubility enhancement and encapsulation of hydrophobic drugs. However, the major constraint with these approaches is the lack of their applicability to a wider variety of hydrophobic drugs (Lakshmi and Ashwini, 2010). During the last several years, nano-polymeric carrier “Dendrimer” has been widely studied in attempts to address the problems associated with the solubilization of hydrophobic drug molecules. Dendrimers not only are able to overcome the above mentioned limitations of the existing drug solubilization methods, but also offer various added advantages for solubility enhancement.

## Dendrimer: definition, origin, and properties

**Figure 1:**
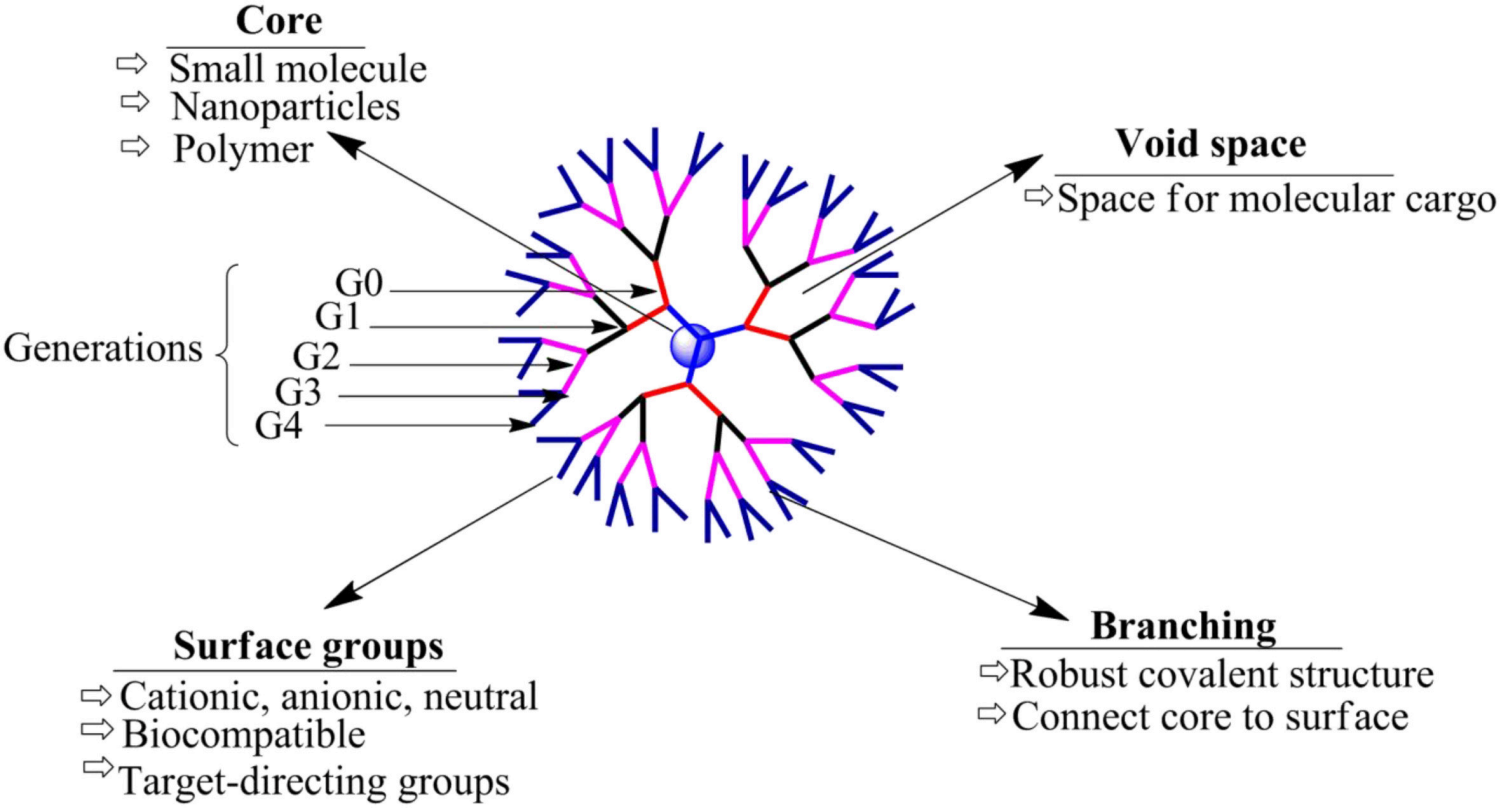
**General representation of the model structure of a dendrimer**.

Dendrimers are large and highly branched polymers, the structures of which are determined by three unique components: (1) the initiator core, (2) the interior layer, made up of repetitive generations (units) connected with initiator core, and (3) the periphery (surface functionality). Representation of a typical dendrimer is shown in Figure 1, which includes all important parts, i.e., core, branches and generations (Tomalia et al., 1985). The first dendrimer (by divergent method) was synthesized by Buhleier et al. (1978). Denkewalter and co-worker at Allied Corporation synthesized polylysine dendrimers in 1981. Although, the first dendrimer was synthesized earlier, the term “dendrimer” was first introduced in 1983. Donald Tomalia and co-workers synthesized and characterized these hyper-branched polymers in 1983 at Dow Chemicals and these molecules were named as “Dendrimer” (Tomalia et al., 1985). Later on dendrimer was synthesized using convergent method for the first time by Hawker and Fréchet (1990).

The first synthesized dendrimers were polyamidoamines (PAMAMs) introduced in 1980s; however, various other dendrimers including poly(propylene imine) dendrimers (PPI), tecto dendrimer, and amphiphilic dendrimers, were synthesized in the later years (Alper, 1991). PAMAM dendrimers are also called as “Starburst®” dendrimers, a trademark of the Dow Chemicals Company (Tomalia et al., 1990). The term “dendrimer” comes from the word “Dendron,” which means a tree. Newkome's group independently reported synthesis of analogous macromolecules, and termed those as “arborols,” which is derived from a Latin word “arbor,” which again means a tree. Another term used for this highly branched polymer structure is “cascade molecule.” Although these hyper-branched molecules have multiple names, “dendrimer” is the most commonly used term (Buhleier et al., 1978; Newkome et al., 1985).

Dendrimer is a synthesized, multi-branched polymeric composition where the branches of the polymer originate from the core. Unique characteristics of dendrimers include their uniformly dispersed design, relatively spherical shape, adaptable surface composition, multi-valency, aqueous-solubility, and available hydrophobic pockets/cavities at the interior which can encapsulate hydrophobes (Esfand and Tomalia, 2001; Svenson and Tomalia, 2005). PAMAM are the most widely used dendrimers (Malik et al., 1999). Dendrimer can be easily tailored at the surface as well as at the interior layers which makes it a versatile host for encapsulation, complexation, conjugation and finally the delivery of a variety of therapeutic molecules. Dendrimers are prepared by a repetitive synthesis process which correspondingly increases the generations and determines the physico-chemical characteristics of the dendrimer product (Tomalia et al., 1985; Newkome et al., 1991).

Dendrimers are unique hyper-branched material with great versatility. They differ from conventional linear natured polymers in that dendrimers have compact and globular structure which is not compressible, and have a spherical shape in contrast to the non-compact, compressible and irregular architecture of linear polymers (Tomalia et al., 1985; Kiefer and Tomalia, 1989; Tomalia et al., 1990). A comparison between linear and hyper-branched polymer is summarized in Table 1. In general, lower generation dendrimers possess open structure, and become globular, compact and dense with increasing generations. Moreover, dendrimers are found to be analogous to some biological structures; for example, insulin, cytochrome C, hemoglobin, prealbumin, and hemerythrin closely match with ammonia core PAMAM G3 (3.1 nm in diameter), G4 (4 nm), G5 (5.3 nm), G6 (6.7 nm), and G7 (8 nm) dendrimers, respectively, in terms of dimensional size and the shape (Tomalia et al., 2003; Tomalia, 2004).

**Table 1:** **Properties of dendrimers in comparison to linear polymers (Fréchet, 1994; Fischer and Vögtle, 1999; Inoue, 2000; Gautam et al., 2015)**.

**Property**	**Dendrimer**	**Linear polymer**
Structure	Compact and Globular	Not Compact
Shape	Spherical	Random Coil
Architecture	Regular	Irregular
Synthesis	Stepwise growth	Single step poly condensation
Crystallinity	Non-crystalline and amorphous	Semi crystalline/crystalline
Aqueous solubility	High	Low
Non-polar solubility	High	Low
Compressibility	Low	High

## Drug-dendrimer interactions

**Figure 2:**
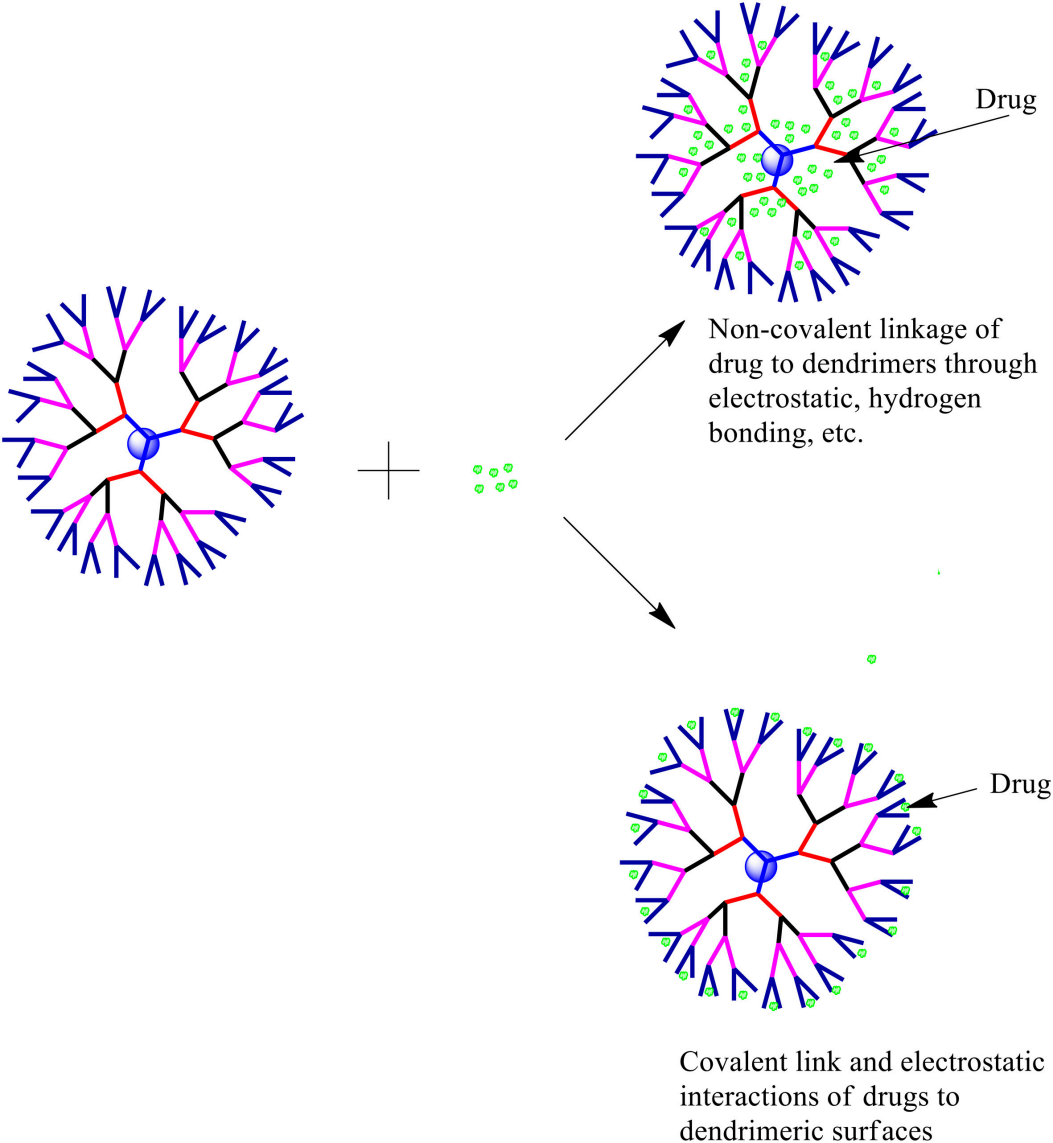
**Possible drug-dendrimer interactions**.

The solubility enhancement property of dendrimers has encouraged researchers to understand the possible dendrimer-drug interactions. Several types of drug-dendrimer interactions have been explored so far, which can be broadly divided into two categories—one is the entrapment/encapsulation of drugs/APIs inside the dendritic structure and the other one is the interaction of the drug and the periphery of the dendrimer. While the former one involves non-covalent forces including hydrogen bonds, hydrophobic interactions and electrostatic interactions, the latter one involves covalent bond formation. Various types of drug-dendrimer interactions are shown in Figure 2.

### Drug encapsulation in dendrimeric cavity

The internal architecture of a dendrimer is usually hydrophobic due to hydrophobic interactions and hydrogen bond formations and is suitable for encapsulating hydrophobic drugs/bio-actives (Medina and El-Sayed, 2009). Higher generation dendrimers have more capacity (in turn more space) to encapsulate hydrophobic moieties. Though, with rising number of branching and surface groups, the exposure of the interior sections of the dendrimer to the continuous vehicle phase significantly reduces due to the “de Gennes dense packing” and structural-folding (Kiefer and Tomalia, 1989; Recker et al., 2000; Boas et al., 2001; Morgan et al., 2003; Medina and El-Sayed, 2009; Svenson and Tomalia, 2012).

The intensity of the interactive forces between the neighbor functional groups within the molecule along with the properties of the bulk solution (i.e., pH, polarity, temperature, etc.) play a major role in the “de Gennes dense packing” occurrence. These properties of dendrimer can be exploited to modulate the encapsulation and release of the drug molecules from the dendritic structures (Jansen et al., 1995; Boas et al., 2001). Although non-covalent drug complexation/entrapment in dendrimers is the preferred technique for solublization of several drugs, such an approach has its limitations too; for example, after exposure to biological fluids the drug-dendrimer structure can fail to control the release of the drug from the dendrimer pockets/cavities (Kojima et al., 2000; Liu, 2008; Kesharwani et al., 2014) due to insufficient interactive forces between the drug and the dendrimer molecules (Wolinsky and Grinstaff, 2008). However, if the dumping of the encapsulated drug can be minimized or avoided, physical encapsulation of drugs in dendrimeric cavities is an attractive approach for solubilization of hydrophobic drug molecules.

### Drug-conjugation

Terminal-functional groups of a dendrimer provide sites for covalent conjugation of diagnostic, therapeutic and biological molecules. Such a conjugation can be used to develop a prodrug. The linker/spacer can be used in preparation of the drug-dendrimer conjugation to transform macromolecular functionality and release profile of the conjugated entities (Svenson, 2009). These linkers such as ester and amide groups, acid labile acyl hydrazone or cis-aconityl groups, and disulfide bridges covalently attach to the drugs and dendrimers and thus conjugate the cargo and the carrier. Studies have confirmed the role of linkers in *in vivo* stability of dendrimer-drug conjugates (Najlah et al., 2006, 2007). Several attempts have been made to attach drug molecules with dendrimers through disulfide linkages, which can be modulated by glutathione inside the cells to control the release of the drug from the complex (Kobayashi and Brechbiel, 2003; Svenson and Tomalia, 2012). Dendrimers have also been successfully used for diagnosis (Wiener et al., 1994; Kobayashi and Brechbiel, 2003; Mintzer and Grinstaff, 2011; Svenson and Tomalia, 2012). Dendrimer based contrast agents offer tissue specificity, do not suffer from rapid excretion, and require smaller dose, and hence are advantageous in comparison to the conventional diagnostic agents. In addition, dendrimers have been successfully conjugated and delivered with various immunogenic proteins for the purpose of vaccination (Tam, 1988; Bay et al., 1997; Ota et al., 2002).

## Factors influencing drug solubilization and delivery

**Figure 3:**
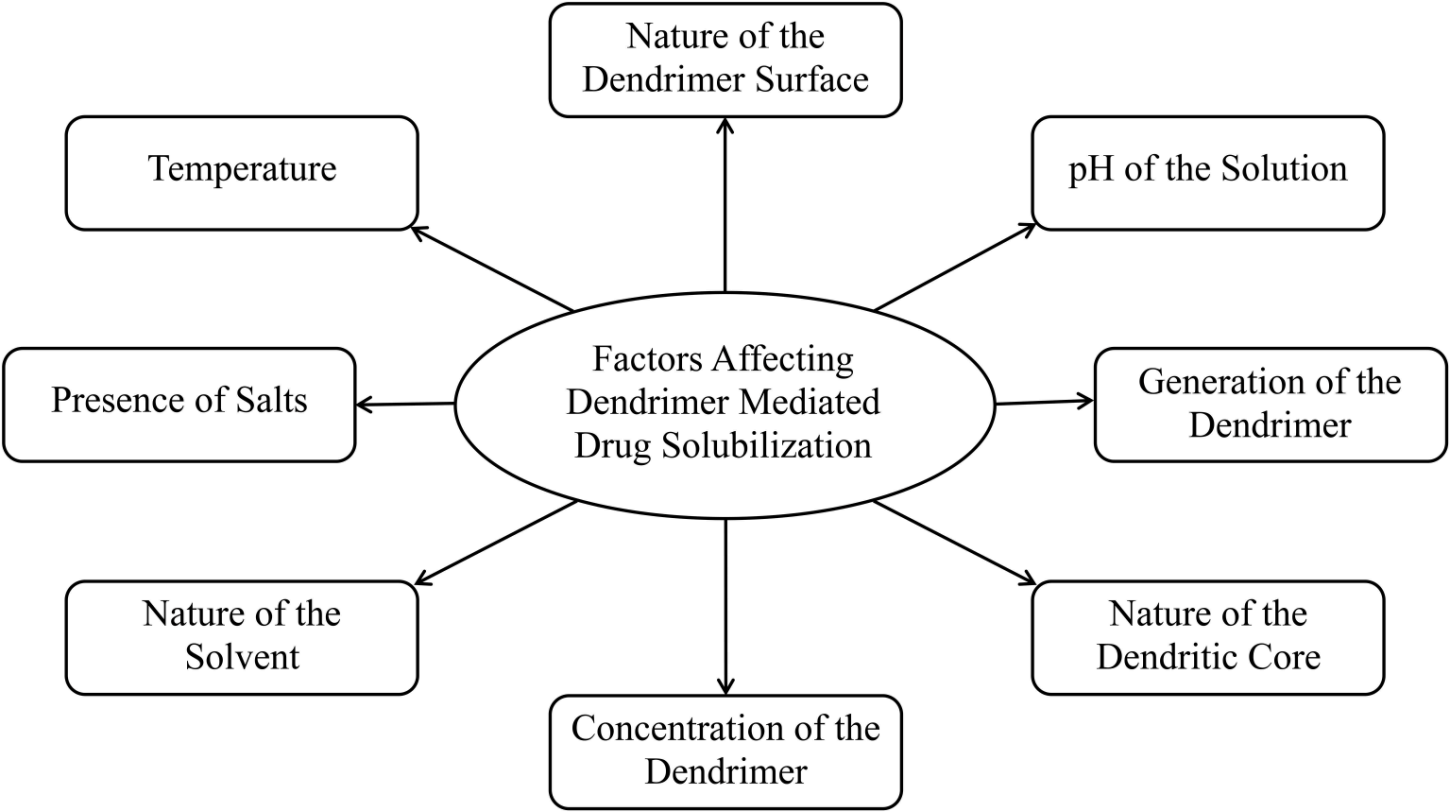
**Factors affecting dendrimer mediated drug solubilization**.

Open dendritic architecture of dendrimer provides opportunities for interaction with labile or poorly soluble drugs. Numerous investigators explored and optimized the encapsulation and complexation of various drug molecules using dendrimers. Although, dendrimer mediated drug solublization and delivery is an attractive approach applicable to a wide variety of drugs, there are several factors—including, but not limited to, the pH of the solution, dendrimer generation, dendrimer surface, nature of the dendritic core, and the concentration of the dendrimer in the solution—which can affect this solubilization approach, and the outcome (Figure 3).

### pH

Although the interaction of hydrophobes and surface amine groups (internal, peripheral, or tertiary) of dendrimers plays a crucial role in the improvement of drug solubility and the drug delivery, protonation behavior and pH of the dendrimers are very important too. The periphery as well as the interior of the dendrimer is influenced by the pH of the medium (Maiti et al., 2005). Asthana et al. reported a proportional increase in the loading efficiency of flurbiprofen in water using PAMAM dendrimer (G4)—greatest at pH 10, intermediate at pH 7, and the lowest at pH 2 (Asthana et al., 2005). Several studies have reported an important role of pH in the solubility of various drugs including ibuprofen (Milhem et al., 2000), ketoprofen and nicotinic acid (Yiyun and Tongwen, 2005a,b), when used in combination with PAMAM dendrimers. It has been reported that the possible mechanisms behind the solubility enhancement of ibuprofen and ketoprofen are their ionization and electrostatic interactions with dendrimer surface amines which are pH dependent (Milhem et al., 2000; Yiyun and Tongwen, 2005a,b). These studies clearly emphasize that the pH of the medium is an important parameter that can significantly affect the dendrimer assisted drug solubilization. The protonation state of dendrimer at the given pH must be considered when using dendrimer mediated drug solubilization approach.

### Generation of the dendrimer

As the generation of a dendrimer increases, its size increases accordingly, and with increasing size the shape of the dendrimer becomes more spherical and defined. When the dendrimers are smaller in size, their structure is relatively loose and less defined, while high-generation dendrimers adopt a globular architecture which can carry multiple drug molecules on their surface as well as in the cavities inside the dendritic structure. Increase in generation of dendrimers increases the interior voids and hence it increases the drug solublization properties. Kaanumalle et al. reported 5-, 8-, and 24-fold increase in aqueous solubility of pyrene using first, second, and third generations of poly(alkyl aryl ether) dendrimers, respectively (Kaanumalle et al., 2004). Similarly, several researchers reported dendrimer-mediated solublization proportional to the concentration of dendrimers in the system (Pistolis and Malliaris, 2002; Devarakonda et al., 2004; Yiyun and Tongwen, 2005b). In general, dendrimers of lower generation are preferred over those of higher generations as these are less cytotoxic, less immunogenic and more biocompatible (Duncan and Izzo, 2005). In the past, we attempted to explore the capability of dendrimer mediated solubility enhancement of three different hydrophobic drug molecules including famotidine, indomethacin and amphotericin B. The study concluded that the pH and protonation status play a key role in solubility enhancement of hydrophobic drugs (Gupta et al., 2007).

### Nature of dendrimer surface

The cytotoxicity of the dendrimer is based on the chemistry of its core and the surface. For example, the cytotoxicity of cationic dendrimers on Clone-9 cells on a melamine-based dendrimer library including amine, guanidine, carboxylate, sulfonate, and phosphonate modification, inferred that positively charged dendrimers induce greater cytotoxicity in comparison to negatively charged dendrimers (Chen et al., 2004). When the PAMAM dendrimer surface was modified using amino acids including lysine and arginine its cytotoxicity was significantly higher in comparison to the unmodified PAMAM (Choi et al., 2004). Malik et al. reported that dendrimers with carboxylic acid groups on the surface—hence a negative charge—were non-toxic in various cell lines at or below 5 mg/mL concentration (Malik et al., 2000). While at higher generations, dendrimer core becomes less accessible due to the branches and the surface groups of the structure, and the surface of the dendrimer becomes less accessible due to steric hindrance, small-generational dendrimers have loose structure and hence an accessible core along with available surface groups for interactions. Various strategies including covering the dendrimer surface with polyethylene glycol have been explored to address the toxicity issue associated with the dendrimer surface (Malik et al., 2000; Ihre et al., 2002; Xyloyiannis et al., 2003). This biocompatible surface provided by the molecules such as PEG further improves the stealth nature of dendrimers in systemic circulation (Singh, 2005).

### Dendritic core properties

The interior architecture of a dendrimer is established by the nature of the core, the branching molecules and the terminal groups. A comprehensive and extended core ensures well-designed cavities/pockets and provides better flexibility to the unimolecular dendritic architecture (Uppuluri et al., 2000). Effects of larger interior cavities because of selection of an appropriate core was apparent from the augmentation in hydrophilicity of pyrene reported by Hawker et al. (1993) and Liu (2008). The solubilization of pyrene increased by 120-fold when a dendritic architecture synthesized using 3,5-dihydroxy benzyl alcohol as the building block was used to improve the solubility (Hawker et al., 1993). Similarly, analogous unimolecular micelles were synthesized using 4,4-bis(4-hydroxyphenyl) pentanol as a substitute of 3,5-dihydroxybenzyl alcohol which led to 356-fold increase in the solubility of pyrene (Liu, 2008).

### Concentration of dendrimer

Studies have reported amplification in dendrimer-mediated drug solubilization when the concentration of the dendrimers in the solution was increased (Lach and Cohen, 1963; La et al., 1996; Liu, 2008). Although it is important that the dendrimer concentration be appropriately selected while bearing in mind the potential toxicity and bioincompatibility issues associated with dendrimers (Kolhe et al., 2006). As the dendrimer surface has amine terminal groups which lead to cationic toxicity at higher concentration, there is a maximum safe dose for dendrimers which is determined by its composition. If used at an appropriately selected concentration below the toxic levels, these carriers are excellent solubilizers. Chauhan et al. studied the solubility of indomethacin with different concentrations of NH_2_ and OH-terminated G4-PAMAM dendrimers and observed a linear enhancement in the drug solubility with increasing concentration of dendrimers, in contrast to phase solubility studies (Chauhan et al., 2003). Similar Trends were observed by Patel et al. when the solubility of aceclofenac in the presence of various concentrations of G-0 and G-3 PAMAM dendrimers was investigated (Patel et al., 2011). For both the generations, solubility of aceclofenac increased in a linear fashion with an increasing concentration of dendrimer.

### Nature of the solvent

Solvents have ability to solvate the dendrimer, while investigating the conformation of the dendrimers. Generally, dendrimers intellect great level of back-folding with diminished solvation property. In comparison to higher generation dendrimers, low generation dendrimers are more flexible with their structures and hence are more susceptible to back-folding when in an environment of poor solvation. When the solvent is acidic in nature for a basic dendrimer, or vice versa, there could be a significant change in the dendrimer conformation, due to the interaction between the dendrimer groups and the solvent molecules. Chloroform, being a weakly acidic solvent, shows significant hydrogen bonding with the NH_2_ groups at the surface and inner parts of the dendrimer leading to swelling of the dendrimer structure (Chai et al., 2001). Hypothetical and investigational analysis on NH_2_- PPI and NH_2_-PAMAM showed that the vehicles which are not polar in nature lead to superior molecular-density at the dendritic-core due to back-folding, while polar solvents solvate the arms of dendrimer and induce higher molecular-density on the dendrimer exteriors (Gupta et al., 2006a). Back-folding of the polar surface groups may render the hydrophobic dendrimer components unsuitable to the ambience leading to a reduced surface polarity of the dendrimer. These aspects must be considered in achieving dendrimer-mediated solublization (Gupta et al., 2006b).

### Presence of salts

High concentration of salts has a strong effect on charged PPI dendrimers and favors a contracted conformation of dendrimers with a high degree of back-folding. A similar effect is observed with ascending H^+^ ion concentration in the dendrimer solution, and also when the dendrimer is poorly solvated. When the concentration of the salt in the dendrimer solution is lower, the repulsion among the ionized groups on the dendrimer leads to unfolding of the dendritic architecture (Gupta et al., 2007). Tian and Ma studied the effects of multivalent salt ion concentration on the structural conformation of dendrimer and reported that when the salt concentration in the dendrimer solution increased, it led to contraction of the dendritic structure (Tian and Ma, 2010).

### Temperature

In general, heat has a profound effect on the behavior of drug molecule when in solution; especially the solubilization properties of the drug may significantly change with change in temperature. Milhem et al. studied the heat effects on solubilization behavior of Ibuprofen in combination with G4 PAMAM dendrimers at various temperatures including 27°, 35°, 40°, 45°, and 50°C (Milhem et al., 2000). The drug solubility increased with an increase in the temperature; however the possible reasons for the unusual solubility pattern were not discussed in the report. Wang et al. studied the capacity of magnetic poly-(methyl acrylate-divinyl benzene) microspheres with amine terminated dendrimers on the surface for adsorbing hexavalent chromium and reported that the adsorption capacity of the microspheres increased with increasing temperature (Wang et al., 2012). However, it is important to note that hexavalent chromium is a heavy metal ion and is not a drug, and adsorption is a different phenomenon than solubilization. The impact of temperature on dendrimer assisted drug solubilization is yet to be fully explored, and further research efforts are warranted to understand the involved mechanisms (Jain and Tekade, 2013).

## Solubilization of existing drugs

During the last few decades dendrimers have proven their usefulness as solubilizers (Table 2). The unique properties of dendrimers including its superior host-guest chemistry, multivalent geometry, high aqueous solubility, high encapsulation efficiency and the adaptable surface architecture makes it an excellent drug solubilizing agent. Usually a generation dependent change in the properties and performance of a dendrimer is observed when used for drug solubilization. As shown in Figure 1, the structure of low-generation dendrimers (G1–G3 generation) are relatively open and asymmetric in shape, and high-generation dendrimers have more compact structure and globular (G4 and above) shape (Gupta et al., 2006a). The globular shape also accounts for the higher loading capacity of the drug inside the structure of dendrimers. In the following section we will discuss the hydrophobic drugs for which the solubility has been improved using various dendrimers. A summary of these drugs have been provided in Table 3.

**Table 2:** **Dendrimer mediated solubility enhancement of drugs**.

**Drug/API**	**Dendrimers used**	**Structure**	**References**
Nifedipine	Amine and Ester-terminated PAMAM dendrimers	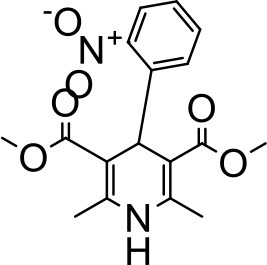	Devarakonda et al., 2004
Artemether	PEGylated lysine dendrimers	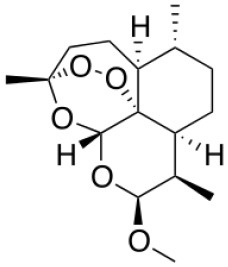	Bhadra et al., 2005
Silicone dioxide	PAMAM dendrimer	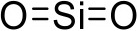	Neofotistou and Demadis, 2004
Nicotinic acid	PAMAM dendrimers	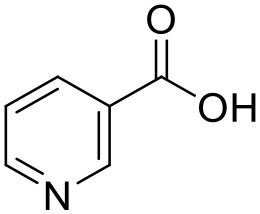	Yiyun and Tongwen, 2005a
Orange dye	Lysine dendrimer	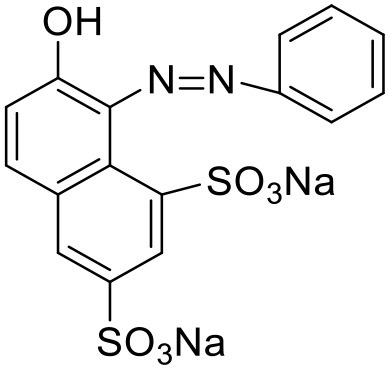	Chapman and Morrison, 1994
Naproxen	PAMAM dendrimers	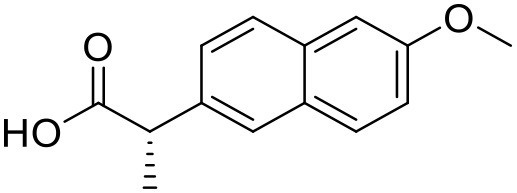	Yiyun and Tongwen, 2005a
Bengal Rose	Polypropylene dendrimer	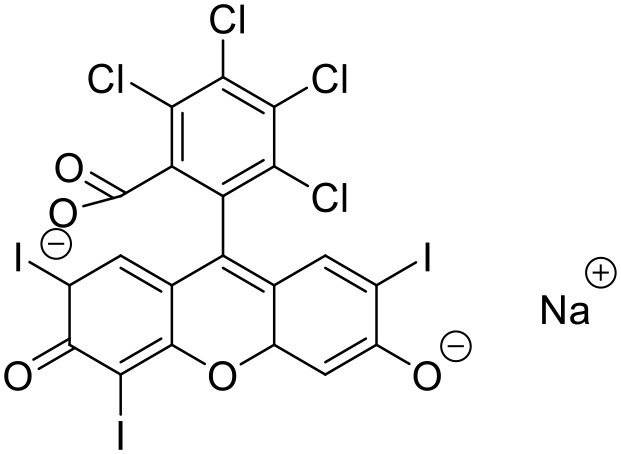	Baars et al., 2000
Niclosamide	PAMAM dendrimers	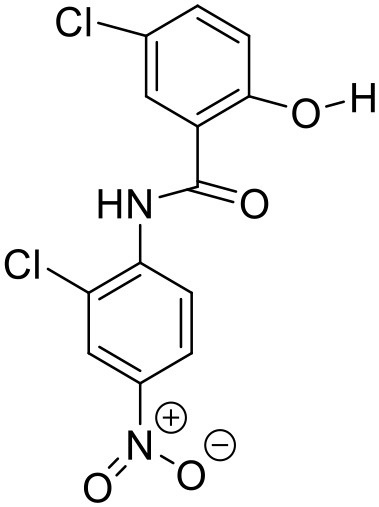	Devarakonda et al., 2005
5-fluorouracil	PEGylated PAMAM dendrimers	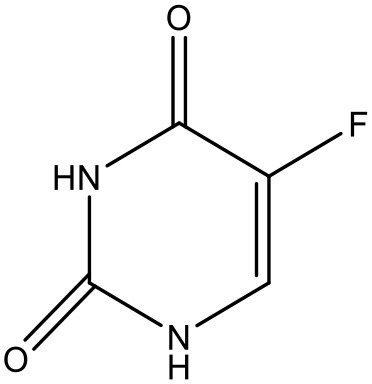	Bhadra et al., 2004
Ibuprofen	PAMAM dendrimers	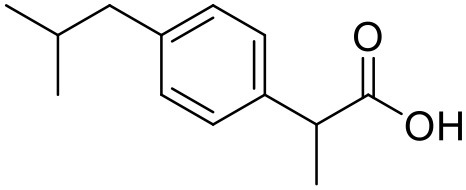	Milhem et al., 2000
Pyrene	Poly(aryl alkyl ether) Dendrimer	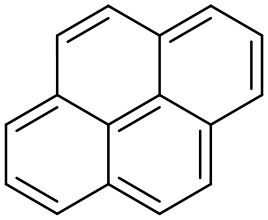	Vutukuri et al., 2004
Pyrene	PEGylated PPI Dendrimers	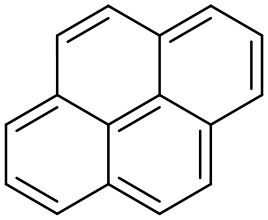	Sideratou et al., 2001
Pyrene	Polyether dendrimer	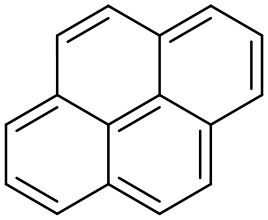	Hawker et al., 1993
Piroxicam	PAMAM dendrimers	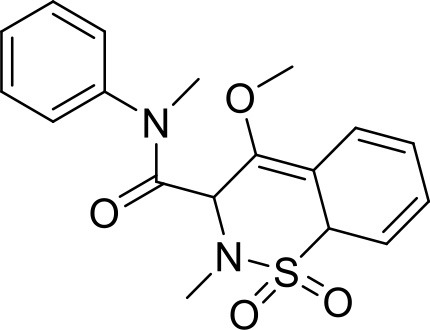	Wiwattanapatapee et al., 1999
Pyrene	Polyether-PEG Dendrimer	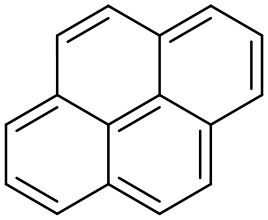	Liu, 2008
Proflavine	Amphiphilic dendrimer	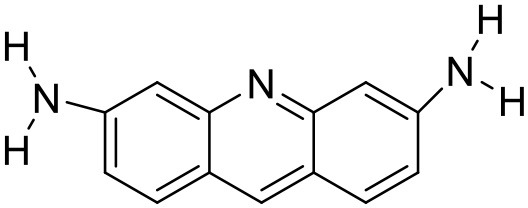	Vutukuri et al., 2004
Pyrene	Polypropylene imine Dendrimer	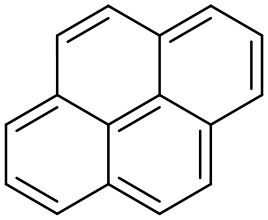	Pistolis and Malliaris, 2002
Mefenamic acid	Citric acid-PEG-citric dendrimer	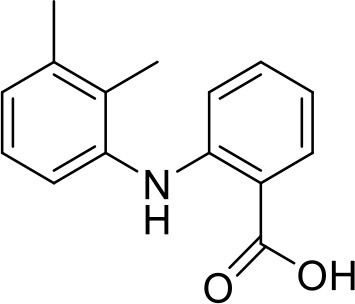	Namazi and Adeli, 2005
Pyrene	PEGylated PAMAM Dendrimers	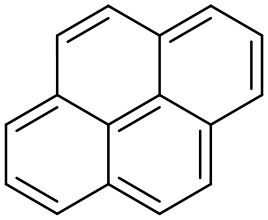	Yang et al., 2004
Propranolol	PAMAM and Lauroyl PAMAM dendrimer	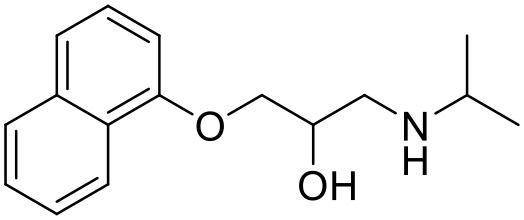	D'emanuel et al., 2004
Paclitaxel	Polyglycerol dendrimer	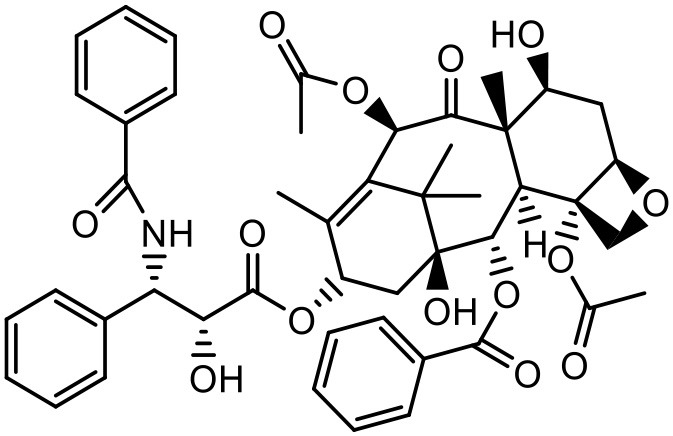	Ooya et al., 2003
Anthracene	Polyether dendrimer	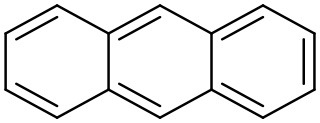	Hawker et al., 1993
Flurbiprofen	PAMAM dendrimers	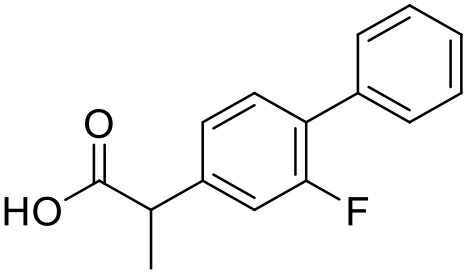	Asthana et al., 2005
Methotrexate	PAMAM dendrimer	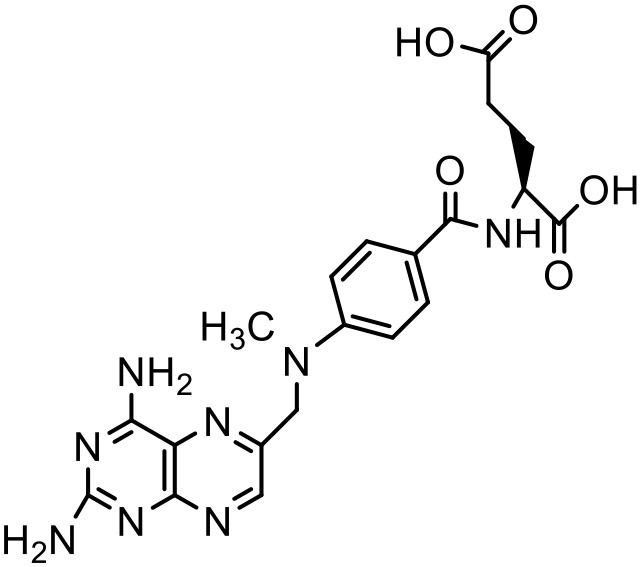	Khopade et al., 2002
Indomethacin	PEG polyethar dendrimers	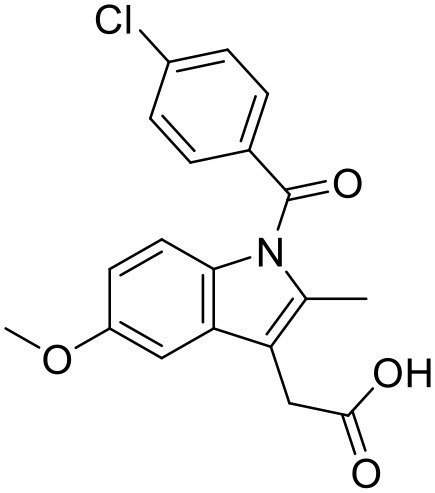	Kwon et al., 1997
Indomethacin	PAMAM –OH dendrimers	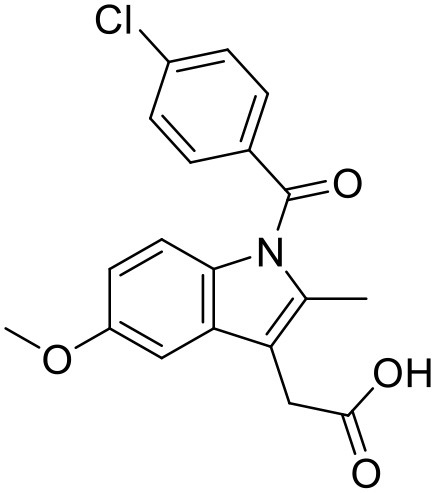	Chauhan et al., 2003
Benzoic acid	Hhydroxyl-PAMAM dendrimer	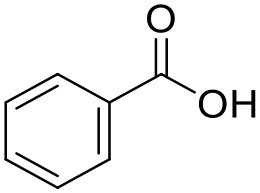	Beezer et al., 2003
Adriamycin	PEG-PAMAM dendrimer	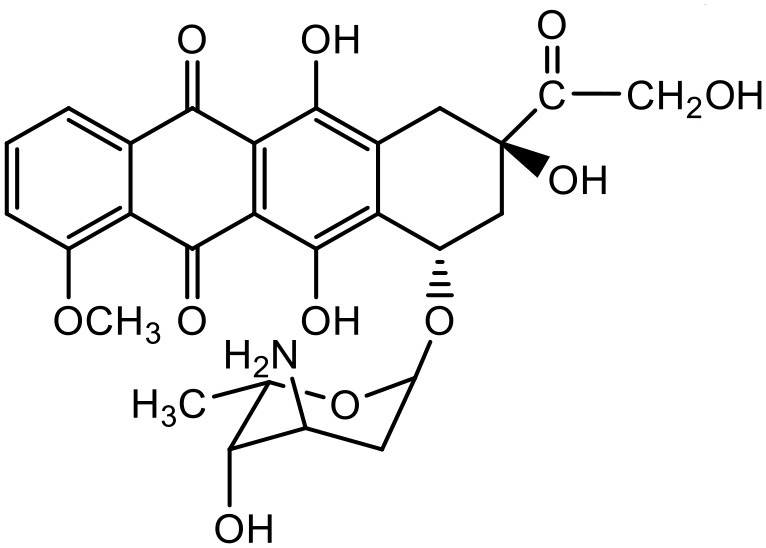	Kojima et al., 2000
Methotrexate	PEG-PAMAM dendrimer	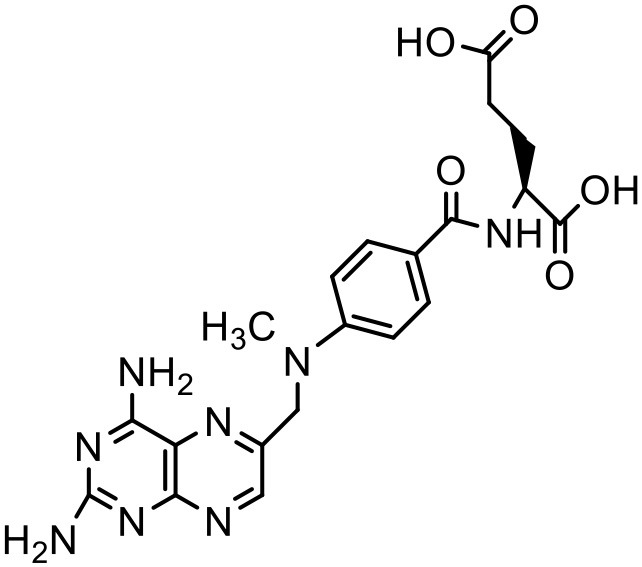	Kojima et al., 2000

**Table 3:** **Recent reports of solubilization of various hydrophobic drugs using dendrimers**.

**Drug/API**	**Dendrimers used**	**Dendrimer generation**	**Structure**	**References**
Aceclofenac	PAMAM dendrimer	G0, G3	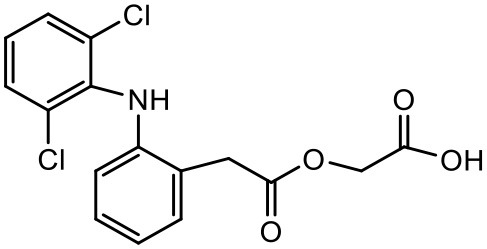	Patel et al., 2011
Amphotericin	PAMAM dendrimer	G1–G3	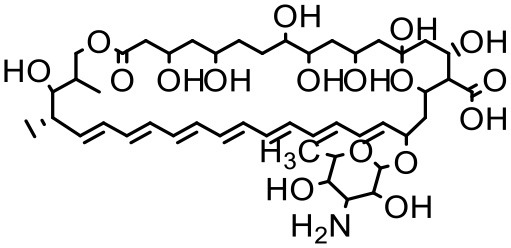	Jose and Charyulu, 2015
Albendazole	PAMAM dendrimers	G3, G3OH, G2.5 and G3.5	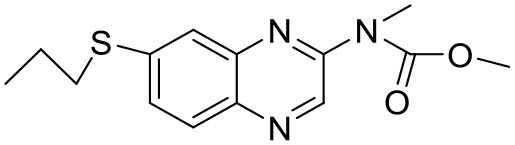	Fernández et al., 2011
Silybin	PAMAM dendrimer	G1.5, G2, G2.5, and G3	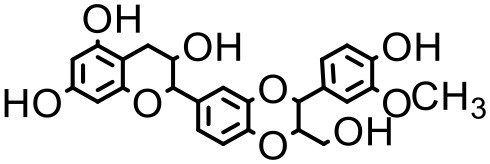	Huang et al., 2011
Docetaxel	Dendrimer–TPGS mixed micelles	G4	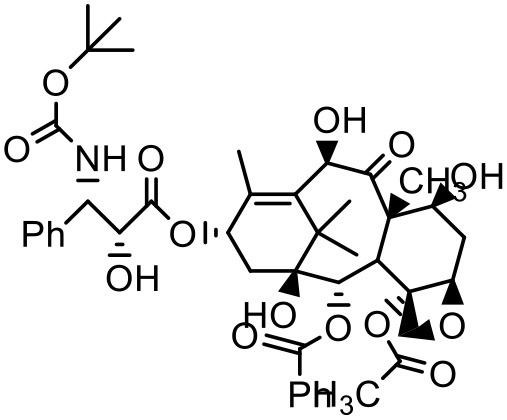	Pooja et al., 2014
Paclitaxel	Dendrimer–TPGS mixed micelles	G4	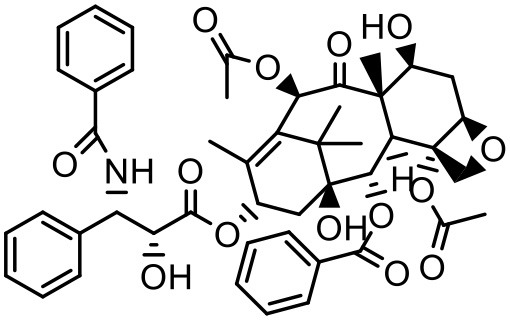	Pooja et al., 2014
Simvastatin	PAMAM dendrimer	G4-PAMAM–NH2, G4-PAMAM–OH and G4-PAMAM–PEG	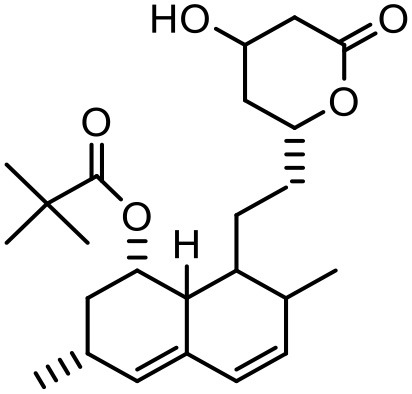	Kulhari et al., 2011
Haloperidol	PAMAM 1,4-diaminobutane Core, -NH_2_	G5	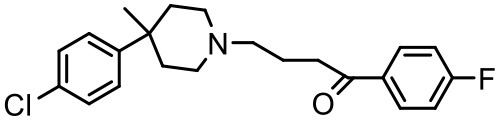	Katare et al., 2015
Risperidone	PAMAM dendrimers	G4	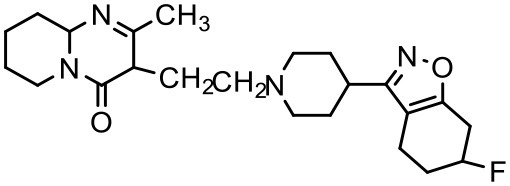	Prieto et al., 2011
Fluorouracil	Poly(amidoamine) dendrimer(PAMAM-NH2 G4) complex	G4	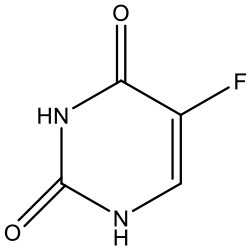	Buczkowski et al., 2011
Beclomethasone dipropionate	PAMAM dendrimers	G3, G4 and G4	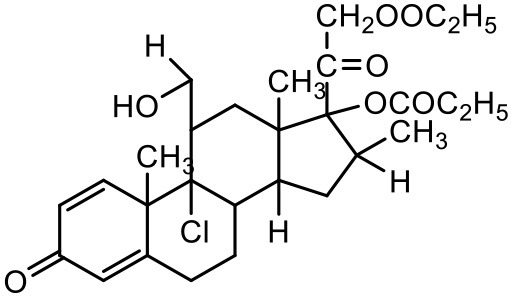	Nasr et al., 2014
Candesartan	Polyamidoamine (PAMAM) dendrimers	G4	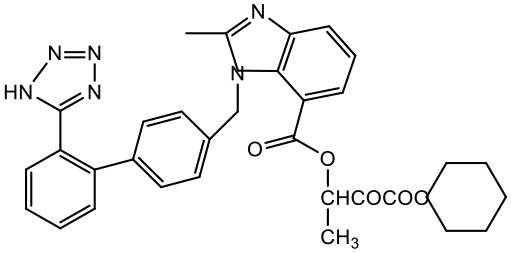	Gautam and Verma, 2012
Paclitaxel	Poly(butylene oxide) (B)–poly(ethylene oxide) (E) block copolymer B16E42 (BE) with a G2 PAMAM dendrimer	G2	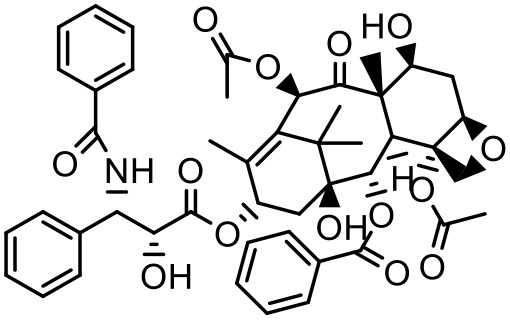	Zhou et al., 2013
Ketoprofen	PPO@PAMAM	G0–G5	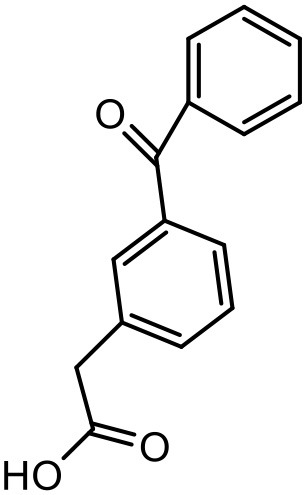	Koc and Mehmet, 2013
Diflunisal	PPO@PAMAM	G0–G5	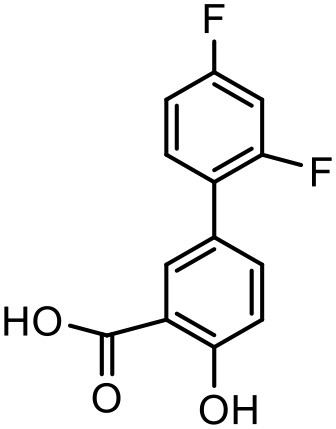	Koc and Mehmet, 2013
Ibuprofen	PPO@PAMAM	G0–G5	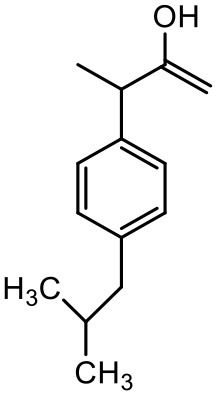	Koc and Mehmet, 2013
Imatinib	(Propylene) imine dendrimers	G5	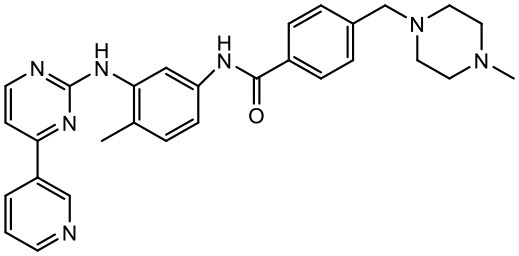	Karthikeyan and Vijayarajkumar, 2015
Rifampicin	PAMAM	G4	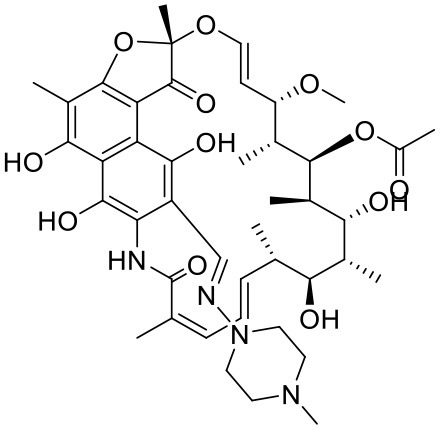	Bellini et al., 2015

### Aceclofenac

Aceclofenac is a phenyl-acetic acid derivative and belongs to the class of non-steroidal anti-inflammatory drugs (NSAIDs), used in the management of osteoarthritis, rheumatoid arthritis, and ankylosing spondylitis. The use of NSAIDs is limited by their toxicity. Most common adverse effects of NSAIDs are gastrointestinal discomfort, nausea, and diarrhea (Polisson, 1996). It is well reported that many of the NSAIDs can cause damage to esophagus, stomach, duodenum, small intestine and large intestine upon oral administration at higher doses (McCarthy, 1999). Morevoer, as NSAIDs have poor aqueous solubility, it is generally challenging to develop suitable topical or parenteral formulations (Lagrange et al., 2000). Aceclofenac is practically insoluble in water. Reported studies have shown that the solubility of aceclofenac can be significantly enhanced using G0 PAMAM dendrimers (Patel et al., 2011). In the study Patel et al., the effect of pH conditions, concentration, temperature and the generation of dendrimers were investigated and it was concluded that the solubility enhancement was concentration dependent. The effect of dendrimer generation at constant pH increased the solubility in an order of G3 > G2 > G1 > G0. Effect of dendrimer pH on the solubility improvement of aceclofenac could be due to an electrostatic interaction among NH_2_ groups of dendrimer and COOH group of the drug. The aceclofenac solubility was found to be inversely proportional to the temperature of dendrimer solution (Patel et al., 2011).

### Amphotericin-B

Amphotericin-B (AmB) is a polyene antibiotic, commonly used for systemic fungal infections. The clinical use of AmB is limited, because it has poor aqueous solubility, and it induces nephrotoxicity, which can cause permanent renal impairment (Jose and Charyulu, 2015). Jose and Charyulu studied the effects pf PAMAM dendrimers on aqueous solubility of AmB. The results showed an enhancement in solubility of AmB when in combination with PAMAM dendrimers (G1). AmB solubility in the dendrimer solutions improved in a relatively linear way with increasing concentration of dendrimer. The solubility enhancement of AmB was attributed to the internal cavities of dendrimers which can encapsulate AmB molecules (Jose and Charyulu, 2015). Two important parameters of dendrimer mediated solubility improvement are the total area for cargo and the number of amino groups available on the dendrimer particles; hence a higher generation of PAMAM dendrimer has a greater capability to adsorb and interact with AmB molecules than a lower generation one. A reported study has shown that the solubility of AmB increases as the generation of dendrimers increases (Hu et al., 2004). The results showed that an increase in solubility of drug was completely dependent on the concentration and generation of the dendrimer. Dendrimers are considered as static unimolecular micelles and their micellar structure remains stable even at higher concentrations of solvents (Newkome et al., 1991; Hawker et al., 1993; Stevelmens et al., 1996). The effect of pH on the solubility of AmB was reported in the order of 7.4 > 10.0 > 4.0 in PAMAM G3 dendrimeric formulations (Jose and Charyulu, 2015). At the pH of 4, only slight increase of solubility was observed when compared to that at higher pH, i.e., 7 and 10. The reason behind this increase in solubility was the interaction between surface amine groups of dendrimers and functional groups of drug molecule. However, it is important to note that the drug interaction with the dendrimer surface, and hence its solubility can change with change in the pH of the solution, as discussed earlier (Hu et al., 2004; Jose and Charyulu, 2015). Our previous study has reported improved solubility of AmB when used with PPI dendrimer (Gupta et al., 2007).

### Albendazole

Albendazole (ABZ) is a broad spectrum anthelmintic agent (Casulli et al., 2006) widely used for the management of cerebral cysticercosis—a common public health issue (Zongde et al., 2005). Additionally, ABZ is under investigation in malignancy treatment (Zhao et al., 2008). Limited aqueous solubility is one of the main challenges associated with ABZ (0.61 μg/mL), which is responsible for its poor bioavailability (Wu et al., 2005). Fernández et al. used ethylenediamine core PAMAM dendrimers in an attempt to improve the aqueous solubility of ABZ. G3 PAMAM dendrimers (-NH_2_ terminated, –OH terminated) and carboxylate terminal 0.5G, 2.5G, and 3.5G PAMAM dendrimers, were used for investigating their effect on solubilization of ABZ (Fernández et al., 2011). Studies suggest that the mechanism behind the solubilizing effect of dendrimers on ABZ could be ionic interactions, hydrophobic drug-dendrimer interactions and hydrogen bonding (Gupta et al., 2006b). Primary NH_2_ on the surface and tertiary NH_2_ at the interior sites of PAMAM dendrimers can serve as hydrogen bond donors and acceptors, respectively. ABZ also has proton-giver as well as receiver groups, so an intra-molecular hydrogen bond formation occurs between the proton of the amine group on the aromatic ring and the carbonyl of the carbamoyl moiety of ABZ. The outcomes obtained by Fernández et al. showed that the dendrimers have the ability to improve the aqueous solubility of ABZ and other hydrophobic drugs. It was concluded that both specific hydrogen bonds and lipophilic interactions lead to improvement in the solubility of ABZ. The change in solubility augmentation with the dendrimer types could be due to the type of the ABZ-dendrimer interactions, depending on the surface functional groups of dendrimers (Fernández et al., 2011).

### Silybin

Silybin is obtained from the milk thistle plant, *Silybum Marianum*, and has been used for many years as a natural remedy for hepatitis and cirrhosis, and as a hepato-protective agent (Kvasnicka et al., 2003; Tedesco et al., 2004). However, the solubility of silybin is extremely low in both water and oil, and it exhibits poor absorption in the gastrointestinal tract, which results in very low bioavailability (Barzaghi et al., 1990; Morazzoni et al., 1992). Huang et al. investigated various generations of PAMAM for solubilization enhancement of silybin at different pH conditions (Huang et al., 2011). Dendrimer concentration at 37°C was found to have a positively linear correlation with the aqueous solubility of silybin. The improvement in solubility was due to an electrostatic interaction between the dendrimer surface groups and the silybin molecules. The study also investigated the effects of pH conditions on drug solubilization. The drug solubility in dendrimer solution was found to be the highest at pH 10.0 and the lowest at pH 4.0. Low solubility of the drug at lower pH could be due to its unionized status which may not allow it to interact with the amine groups on the dendrimer surface. The study also investigated *in vivo* performance of the drug dendrimer complex, and reported a controlled release of the drug from the complex, and improved bioavailability. In a recent study by Diaz et al. PEGylated G-4 PAMAM dendrimers led to 5-fold enhancement in the solubility of silybin. The study found that the drug was forming complexation with the dendrimer groups as well as with the PEG groups on the dendrimer surface (Diaz et al., 2017).

### Docetaxel

Docetaxel (DTX) is one of the most commonly used drugs for the treatment of cancer due to its high efficacy and broad spectrum anti-cancer activity. This anti-cancer drug has shown high cytotoxicity against various cancers including those of breast, lung, brain, pancreas, prostate, ovaries as well as head and neck (Ringel and Horwitz, 1991; Horwitz, 1992). The development of a drug delivery system for DTX is still a challenge for pharmaceutical researchers, because of its high lipophilicity and inadequate aqueous solubility (Hardman et al., 1996). The solubility of DTX in water is 3–5 μg/mL (Mathew et al., 1992; Ali et al., 1997; Zaske et al., 2001; Hamada et al., 2006; Du et al., 2007). The marketed formulation of DTX, Taxotere® is formulated with 13% of ethanol and polysorbate-80 and causes anaphylactic hypersensitivity, hemolysis and cholestasis (Weiss et al., 1990; Bissery et al., 1992; Ellis et al., 1996; Loos et al., 2003; Marupudi et al., 2007). Pooja et al. used PAMAM with d-α-tocopherol-PEG-succinate (TPGS) mixed-micelles to improve the solubility of DTX. PAMAM dendrimers with diaminobutane (DAB) core with TPGS mixed micelles were prepared by solvent casting method. Different ratios of dendrimers and TPGS were used for determination of the effect on the solubility of taxanes. At equal mass ratio of dendrimer and TPGS (1:1), the DTX solubility in water was found to be 97.48 ± 2.68 μg/mL with encapsulation efficiency of 44.62. As the TPGS concentration increased (low D/T ratio, 1:2), the DTX solubility was increased to 116.67 μg/mL, with DTX encapsulation of 55.59%. The effect of pH on solubility and encapsulation of DTX in dendrimer–TPGS mixed micelles was studied by keeping dendrimer and TPGS at constant ratio of 1:2. Solubility and drug encapsulation of DTX was not significantly (*p* > 0.05) changed with the alteration in pH. DTX solubility at different pH was as follows: 107.32 μg/mL at pH 5; 103.06 μg/mL at pH 7; and 116.67 μg/mL at pH 9. The insignificant (*p* > 0.05) change in the solubility of the drugs can be attributed to the lack of ionization groups in their structures. G4 PAMAM dendrimer structure is reported to be responding to pH conditions because of the presence of primary and tertiary amine groups but its low concentration in mixed micelles could be a reason for the insignificant effect on the solubility of DTX (Pooja et al., 2014).

### Paclitaxel (PTX)

PTX is a widely used anticancer agent due to its superior efficacy against various cancers. Its antitumor action is through inhibition of cellular proliferation by binding to the microtubules of the cells and stabilizing them which leads to prevention of depolymerization (Ringel and Horwitz, 1991; Horwitz, 1992). Due to poor aqueous solubility and high lipophilicity, formulating an appropriate delivery system for PTX has been challenging. Besides, PTX is found to be a substrate for p-glycoprotein and multidrug resistance protein-1 mediated efflux, and hence it has poor efficacy in cells which overexpress p-glycoprotein and multidrug resistance protein-1 (Hardman et al., 1996). PTX solubilizes in the water at a concentration of 0.35–0.7 μg/mL (Weiss et al., 1990; Bissery et al., 1992; Ellis et al., 1996; Loos et al., 2003; Marupudi et al., 2007). The marketed formulation of PTX (Taxol®) contains 50% polyoxyethylated castor oil and 50% ethanol to solubilize PTX but it exhibits many adverse effects such as hypersensitivity, gastrointestinal toxicity and neutropenia. During formulation development of PAMAM dendrimers with diaminobutane (DAB) core–TPGS mixed micelles, different ratios of dendrimers and TPGS were used for the determination of their effect on the solubility of taxanes (PTX) (Pooja et al., 2014). At equal mass ratio of dendrimer and TPGS (1:1), PTX solubility and encapsulation efficiency were 3.40 ± 0.35 μg/mL and 3.01% respectively. Increase in D/T ratio (i.e., more dendrimer concentration) did not significantly (*p* > 0.05) change the solubility and encapsulation of the drug; but as the TPGS concentration was increased (low D/T ratio, 1:2), PTX solubility was observed to be 14.33 μg/mL with 6.87% encapsulation efficiency. Dendrimer–TPGS mixed micelles were prepared and evaluated for their effects on the solubility and anti-cancer activity of PTX. The micelles showed a significant increase in the solubility of PTX. The maximum aqueous solubility and encapsulation of PTX were observed at 1:2 dendrimer to TPGS ratio. The change in pH did not significantly affect the solubility of taxanes (Pooja et al., 2014). Micelles also displayed smaller size (<30 nm), sustained release of encapsulated drug, and good physicochemical stability. During haemolytic studies, micelles showed no haemolysis, which indicated their biocompatibility and utility as carrier system. Anticancer activity of the drug was improved upon its encapsulation in micelles while the toxicity to the healthy cells was lower (Pooja et al., 2014).

In another attempt to increase the aqueous solubility of this taxol derivative, Zhou et al. studied the influence of dendrimer on PTX by using a linear-dendritic block copolymer. The solubilization power of linear-dendritic copolymer (BE-PAMAM) micelles for PTX was investigated in the study. The study found that PTX was 3,700-fold more solubilized upon micellar encapsulation in 2% BE–PAMAM copolymer solution (Zhou et al., 2013).

### Simvastatin

Simvastatin is a synthetically derived lipid-lowering agent from “statins” family which helps with controlling the cholesterol and other fat levels in the body. Major drawbacks associated with this drug are its limited aqueous solubility, poor absorption from the gastrointestinal tract and poor bioavailability (<5%) (Sharma et al., 2009). Kulhari et al. studied simvastatin with dendrimer where the main purpose of the study was to assess the potential of three different G4 PAMAM dendrimers (Kulhari et al., 2011). It was found that the solubilization was highest with PEGylated dendrimers (33-fold), followed by NH_2_ (23-fold) and OH (17.5-fold) terminated dendrimers. Linear correlation was observed between solubility and dendrimer concentration when solubility profile of PEG dendrimer-SMV complex was measured. With 109.04 M (0.4%, w/v) PEGylated dendrimer solutions, the solubility was increased from 33.4 to 1,093.25 μMole/L (33-fold). The mechanism behind the enhancement of solubility is the interaction/complexation between simvastatin and tertiary amines of dendrimer, accessibility of voids for drug entrapment, and hydrogen-bond formation. When the effect of pH was studied, maximum solubilization improvement was observed in the order of pH 10.2 > pH 7 > pH 5. As simvastatin is weakly acidic drug, it remains unionized at low pH (pH 5), and the dendrimer amine groups remain protonated, which result in poor interactions of the drug with the dendrimer. The increase in solubility of simvastatin at higher pH (pH 10), could be due to the strong electrostatic interactions between de-protonated dendrimers and the entirely ionized drug. The study reported that dendrimer-medicated solubility of simvastatin was dependent on the dendrimer surface functionality, dendrimer concentration and pH conditions during the studies (Kulhari et al., 2011).

### Haloperidol

Haloperidol belongs to antipsychotic class of drugs prescribed for the treatment of acute psychosis, schizophrenia, and Tourette's syndrome. It has limited aqueous solubility and an enhancement in its solubilization is warranted for its successful *in vivo* administration (Malone and Waheed, 2009). Katare et al. used dendrimer nanotechnology for brain targeting of haloperidol, via the intranasal, intraperitoneal, and oral routes (Katare et al., 2015). The study reported up to 100-fold increment in haloperidol solubility when used with dendrimers, polysorbate 20, and ethyl alcohol. It was found that the solubility of the drug was improved using dendrimers at a concentration as low as 0.25%, and it was 10 times higher in comparison to the enhancement observed with 20% ethanol, and seven times higher than that with combination of 20% ethanol and 2% polysorbate 20. A cocktail of dendrimers (1%), ethanol (20%), and polysorbate 20 (2%) resulted in an aqueous solution with haloperidol concentration of 1,223 μg/mL, while the aqueous solubility of haloperidol without any solubilizer was found to be 11.5 μg/mL (Katare et al., 2015).

### Risperidone

Risperidone is an antipsychotic drug, which is extensively used in the treatment of autism spectrum disorders (ASD) (Courchesne et al., 2007; Kumar et al., 2008; Marshall et al., 2008). The major drawbacks associated with risperidone are its low aqueous solubility, poor bioavailability, low affinity to protein binding, and extensive first-pass metabolism (Mannens et al., 1994). Since risperidone acts in the brain, it is essential not only to develop a strategy to increase drug bioavailability by circumventing first-pass metabolism but also to achieve desired drug concentration at the site of action, and to minimize the side effects (Kumar et al., 2008). In a recent report, Prieto et al. successfully increased the solubility of risperidone using G4 PAMAM dendrimer (Prieto et al., 2011).

### 5-Fluorouracil

5-Fluorouracil (5-FU) is an antimetabolite fluoropyrimidine analog of the nucleoside pyrimidine with antineoplastic activity. There is a scope for improvement in the aqueous solubility of 5-FU which encouraged (Daniel et al., 2004) to formulate a system enhancing its solubility using dendrimers (Daniel et al., 2003). The increase in 5-FU solubility was investigated and it was found that PAMAM-NH_2_ G4 dendrimer solution shows a linear correlation between 5-FU solubility and dendrimer concentration. The increase in the solubility of 5-FU is due to an electrostatic interaction and hydrogen bond formation between the positively charged ammonium groups and non-dissociated amine groups of the dendrimers, and the 5-FU molecules which have a strongly negative fluorine atoms (Singh, 2005; Buczkowski et al., 2011). Bhadra et al. have reported improved solubility of 5-FU using PEGylated dendrimers (Bhadra et al., 2003).

### Beclomethasone dipropionate

Beclomethasone dipropionate (BDP) is a corticosteroid prescribed for the maintenance treatment of asthma. The drug suffers from limited aqueous solubility (Bakhbakhi et al., 2006; Xu et al., 2012). Attempts toward improving the solubility of BDP have involved the use of liposome formulations which can offer the ability to solubilize the drug and localize its action in the lung for prolonged periods (Saari et al., 1998, 1999; Darwis and Kellaway, 2001). To enhance the solubility of beclomethasone, Nasr et al. complexed BDP with PAMAM dendrimers (Nasr et al., 2014). The complexation depended on generation and concentration of dendrimers and the hydrogen ion concentration of the dispersion medium. It was observed that the amine terminated dendrimers (G3, G4, and G4) formed more stable complexes with BDP compared to the ester terminated (half-generation; G4.5) dendrimers. Generation of the dendrimers also played an important role for enhancing the solubility of BDP. G4 dendrimers showed the highest improvement in the drug solubility indicating that the solubility of this hydrophobic drug directly correlates with the hydrophobicity of the dendrimer core (Nasr et al., 2014).

### Candesartan cilexetil

Candesartan Cilexetil is used for numerous cardiovascular diseases, as a calcium channel blocking agent. The penetrability of this lipophilic drug through biological membranes is determined by water solubility and its lipid-protein partition coefficient in relation to the stratum corneum (Singhai et al., 1997). Gautam and Verma studied the effect of full generation PAMAM (G4) on the solubility of this drug. The study was performed at room temperature using purified water, and the concentration of the drug was found to be 2.63 μg/mL. It was found that the enhancement in solubility of candesartan cilexetil depends upon the concentration of the dendrimers and the highest solubility was found at 10 mg/mL (373-fold). The enhancement in solubility was concentration and generation dependent (Gautam and Verma, 2012).

### Ketoprofen

Ketoprofen is an NSAID used for the treatment of inflammation in rheumatic diseases (Timothy, 2002). Its use by oral administration is restricted due to its limited aqueous solubility. Also its use by topical and parenteral administration is restricted due to its poor aqueous solubility. To enhance the solubility of NSAIDs in water, various attempts were made in the past using different techniques such as addition of surfactants and formation of hydrophilic salts increasing the wettability and micronization of drug particles (Makiko et al., 2000; Vergote et al., 2001). Recently, Koc and Mehmet used a new class of dendrimers [polypropylene oxide cored PAMAM dendrimers (PPO@PAMAM)] to study their potential in enhancing the solubility of ketoprofen (Koc and Mehmet, 2013). Solubility of the drug was improved with increasing core size of the dendrimers. The improvement in the solubility of ketoprofen when in combination with dendrimers can be because of enhancement in the core size and the internal structure of dendrimer molecules, which can facilitate the host-guest interactions and encapsulation of the drug molecules. It was found that the solubility enhancement with PPO@PAMAM was 4-fold higher in comparison to PAMAM with ethylenediamine core (Koc and Mehmet, 2013).

### Diflunisal

Diflunisal is a widely used NSAID (Brooks, 1998). NSAIDs are used as colon cancer chemopreventive agents (Timothy, 2002). The limitation with the diflunisal is its aqueous solubility. To overcome this issue, Koc and Mehmet attempted to enhance the solubility of Diflunisal by using PPO@PAMAM dendrimers at room temperature in buffer solution. From the experimental results, it was concluded that the solubility of diflunisal increased linearly with increasing concentration and generation of dendrimer. Moreover, the size of the core for a constant generation of the dendrimer was found to have a linearly positive correlation with the increment in the drug solubilization. On the basis of this, it can be concluded that with optimized study conditions, PPO@PAMAM dendrimers are potential solubilizers for NSAIDs because of their polypropylene oxide core (Koc and Mehmet, 2013).

### Ibuprofen

Ibuprofen is an NSAID and one of the most commonly used medicine in the world (Laine, 2001). Its efficacy has been long proven for various disease conditions such as arthritis, spondylitis, dysmenorrhea, gout, pericarditis and patent ductus arteriosus (Simon, 1997; Lipton et al., 1998; Schnitzer, 2002; Connolly, 2003; Ong and Seymour, 2003; Kean and Buchanan, 2005; Zochling et al., 2006). However, due to the poor aqueous solubility of ibuprofen, its use in topical and parenteral formulations has been limited. To minimize these limitations Koc and Mehmet aimed to assess the water solubilizing features of PPO@PAMAM dendrimers. The study showed that dendrimers significantly improved the solubility of ibuprofen. At the range of 0–2 mM dendrimer concentration the solubility of the drug increased linearly. Solubility of ibuprofen was increased from 0.12 to 19.06 mg/mL. At higher concentrations of dendrimers, the solubility of this drug was lower because of precipitation of drug-dendrimer complexes. The PPO@PAMAM dendrimer was found to have better solubility enhancing properties than ethylenediamine cored PAMAM dendrimers in the study. The results suggest that the solublization power of dendritic structure can be enhanced by altering the core size and other properties of the dendrimers (Koc and Mehmet, 2013).

### Imatinib mesylate

Imatinib (STI-571) is a low molecular weight, synthetic, 2- phenylaminopyridine derivative, which acts as a selective inhibitor of the BCR-ABL fusion gene product, a tyrosine kinase (Armstrong and Look, 2005). To address the issue of poor aqueous solubility of imatinib, Karthikeyan and Vijayarajkumar studied the drug in combination with PPI dendrimers (5.0G). It was found that the solubility of imatinib was enhanced at pH 7.4 (Karthikeyan and Vijayarajkumar, 2015). A study has reported that the dendrimers could play a role in solubility enhancement as a result of electrostatic interactions, in addition to hydrogen bonding and molecular encapsulation in the cavities of the dendrimeric system (Devarakonda et al., 2005). Studies have reported that 5th generation PEGylated PPI dendrimer increases aqueous solubility of imatinib (Devarakonda et al., 2005; Karthikeyan and Vijayarajkumar, 2015).

### Rifampicin

Rifampicin (RIF) is a bactericidal antibiotic agent from the rifamycin family of drugs (Masters et al., 2005). RIF is reported to be an essential component of the cocktail used in the treatment of tuberculosis (Burman et al., 2001; Petri, 2001). It has limited aqueous solubility which also limits its clinical applications (Agrawal et al., 2004; Sosnik et al., 2010). Bellini et al. investigated RIF in combination with a G4-PAMAM dendrimer and reported that approximately 20 RIF molecules were getting loaded per molecule of the dendrimer (Bellini et al., 2015). The study reported that the drug-dendrimer complex was stable at neutral pH conditions while it was labile at acidic pH conditions where the drug molecules were rapidly releasing from the complex. This unique characteristic of the complex can be exploited for drug targeting for tuberculosis as the environment at the mycobacterium residential site in human body is acidic in nature. Overall, dendrimer offers an advantageous drug carrier and targeting strategy against tuberculosis.

## Current marketing status of dendrimers

Although several research publications have reported the potential of dendrimers for solubilization of hydrophobic drugs, clinical and commercial applications of this approach in the field of drug development and delivery are yet to be proven. Currently there is no marketed pharmaceutical product where dendrimer has been used as a drug solubility enhancer; however, several other dendrimer based products are commercially available for various applications. VivaGel® is a commercialized dendrimer based therapeutic product—formulated as a mucoadhesive gel—used in the treatment of bacterial vaginosis. It is reported to have antiviral as well as antibacterial properties. A phase 3 clinical trial investigating VivaGel® for prevention of recurring bacterial vaginosis has been recently completed^1^, however the data have not been interpreted and the results of the study are not posted, as of April 7, 2017. While VivaGel® is the only dendrimer based approved product with therapeutic properties, several other dendrimer based products including, SuperFect® and PrioFect®—transfection agents for molecular/cellular studies; Stratus® CS—an *in vitro* diagnostic test system for measurement of cardiac biomarkers; Alert Ticket®—a diagnostic system for anthrax detection; Starburst®—commercially available PAMAM dendrimers; and Priostar®—commercially available poly-lysine based dendrimers, are available in the market for their applications in a wide array of disciplines.

## Conclusion

Physicochemical properties are important parameters to consider when it comes to formulation development of a drug entity. Various properties of the drug such as solubility, melting point, and polymorphism can affect the formulation development. Solubility is among the most critical physicochemical attributes of the drug substance, and yet a majority of newly discovered drugs are either hydrophobic or are poorly soluble in water. To overcome this challenge, researchers have been devising newer methods for drug solubilization. Dendrimers possess several unique features in terms of size, shape, branching length, and surface functionality that make them unique carrier for drug solubilization. It is repeatedly shown that dendrimer is a highly effective and multipurpose polymeric architecture for solubility enhancement of various drugs. Improvement of API solubility facilitates the process of formulation development. Various drugs have been developed and investigated in the recent years where dendrimers are used as solubility enhancer for the hydropobic APIs. Upon reviewing the reported literature on dendrimers it is observed that although dendrimers improve the solubility and dissolution of various drugs, the enhancement depends on several physicochemical and experimental conditions such as pH and temperature of the medium, and concentration and surface functional groups of the dendrimers. Dendrimers can improve the solubility of hydrophobic drugs through physical encapsulation or by covalent conjugation. Dendrimers are unimicellar systems and are generally stable upon dilution. Surfactant based micelles are stable only above the critical micellar concentration while dendrimer, being a real molecule and not an assembly, is unaffected by the change in its concentration. Though dendrimers offer unique advantages for solublization and delivery of drugs, the associated cationic toxicity is the major limitation with their use; however, surface engineering of dendrimers using molecules such as PEG can avoid or minimize this issue. In summary, like any other solubilization technology, dendrimer has its limitations too, e.g., it could be toxic beyond certain concentration levels; however, this hyper-branched three dimensional carrier has successfully demonstrated its solubilization and drug carrying capacity for a variety of hydrophobic drug molecules. Dendrimers are expected to have increasing impact on development of hydrophobic drugs in the coming years.

## Author contributions

SC is the key contributor in the preparation of this manuscript and hence is the first author. All other authors contributed equally with preparation and revisions of the draft.

### Conflict of interest statement

The authors declare that the research was conducted in the absence of any commercial or financial relationships that could be construed as a potential conflict of interest.

## Footnotes

^1^http://www.starpharma.com/news/308, accessed on April 4, 2017.

## Abbreviations

5-FU: 5-Fluorouracil
ABZ: Albendazole
AmB: Amphotericin-B
API: Active pharmaceutical ingredient
ASD: Autism spectrum disorders
BCS: Biopharmaceutical classification system
BDDCS: Biopharmaceutics drug disposition classification system
BDP: Beclomethasone dipropionate
DTPA: Diethylene triamine pentaacetic acid
DTX: Docetaxel
GSK: GlaxoSmithKline
LDL: Low-density lipoprotein
NCEs: New chemical entities
NSAID: Non-steroidal anti-inflammatory drug
PAMAM: Polyamidoamine
PPI: Poly(propylene imine)
PPO@PAMAM: Polypropylene oxide cored PAMAM
PTX: Paclitaxel
RIF: Rifampicin
Risp: Risperidone
TPGS: Tocopherol polyethylene glycol succinate.
